# Efficacy and safety of osimertinib plus anlotinib in advanced non‐small‐cell lung cancer patients after drug resistance

**DOI:** 10.1111/1759-7714.14819

**Published:** 2023-02-26

**Authors:** Mingzhao Wang, Jun Zhao, Tong Chen, Xingsheng Hu, Lin Wang, Yuankai Shi, Yutao Liu

**Affiliations:** ^1^ Department of Medical Oncology, National Cancer Center/National Clinical Research Center for Cancer/Cancer Hospital Chinese Academy of Medical Sciences and Peking Union Medical College Beijing China; ^2^ Department of Medical Oncology Yantai Penglai People's Hospital Yantai China

**Keywords:** anlotinib, drug resistance, non‐small‐cell lung cancer, osimertinib, targeted therapy

## Abstract

**Objective:**

To retrospectively analyze the efficacy and safety of osimertinib combined with anlotinib in the treatment of advanced non‐small‐cell lung cancer (NSCLC) after drug resistance, and to explore the related factors affecting the efficacy.

**Methods:**

The clinical data of 34 patients with advanced NSCLC who received osimertinib combined with anlotinib as three or more lines of treatment in the Cancer Hospital, Chinese Academy of Medical Sciences and Peking Union Medical College from June 2019 to March 2022 were collected, and the therapeutic efficacy and safety were analyzed.

**Results:**

A total of 34 advanced NSCLC patients met the inclusion criteria. The objective response rate was 20.6%, the disease response rate was 88.2%, the median overall survival was 19.0 months, and the median progression‐free survival was 6.0 months. The common adverse events were mainly grade 1–2, and only three cases (11.1%) of adverse events were grade 3, including hypertension, proteinuria, and vomiting. No grade 4 or above adverse events were observed. Multivariate Cox regression analysis showed that the Eastern Cooperative Oncology Group Performance Status score and bone metastasis were independent prognostic factors for osimertinib combined with anlotinib as three or more lines of treatment in advanced NSCLC.

**Conclusions:**

Osimertinib combined with anlotinib as three or more lines of treatment in advanced NSCLC was effective and adverse events were tolerable.

## Graphical abstract

Here, we report that osimertinib combined with anlotinib as three or more lines of treatment in advanced non‐small‐cell lung cancer was effective and adverse events were tolerable. The median survival time was 19.0 months and the median progression‐free time was 6.0 months. Multivariate Cox regression analysis showed that the Eastern Cooperative Oncology Group Performance Status score and bone metastasis were independent prognostic factors.

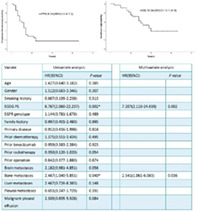

## INTRODUCTION

Lung cancer is the most common cause of cancer‐related death worldwide, accounting for approximately 1.6 million deaths each year, of which non‐small‐cell lung cancer (NSCLC) accounts for about 85%.^1^ The discovery of mutations in the epidermal growth factor receptor (EGFR) gene has ushered in a new era of NSCLC treatment.^2^ Based on the AURA trial for EGFR driver‐positive NSCLC patients, the third‐generation EGFR tyrosine kinase inhibitor (TKI) osimertinib has become the standard of care for patients with T790M mutations who acquired resistance to first‐ and second‐generation EGFR‐TKIs.^3^ In addition, the FLAURA trial showed that the clinical efficacy of osimertinib in the first‐line treatment of advanced NSCLC with EGFR mutations was significantly superior to that of the first‐generation EGFR TKIs.^4^ However, acquired resistance to osimertinib is still inevitably emerging.^5^ Currently, there are very limited options for treatment after resistance to osimertinib. It was reported that resistance to EGFR‐TKI may be associated with enhanced levels of vascular endothelial growth factor (VEGF), and dual inhibition of the VEGFR and EGFR signaling pathways shows potential to overcome resistance to osimertinib.^6^ Anlotinib is an oral novel multitargeted TKI that inhibits angiogenesis and tumor cell proliferation by targeting VEGFR, platelet‐derived growth factor receptor (PDGFR), fibroblast growth factor receptors (FGFRs), tyrosine kinase (c‐Kit).^7^ The purpose of this study was to analyze the efficacy and safety of EFGR T90M‐positive NSCLC patients who received osimertinib combined with anlotinib as the third‐line and post‐third‐line treatments after resistance to osimertinib, and to further explore the potential prognostic factors.

## MATERIALS AND METHODS

### Patients

In this study, a total of 34 patients with advanced NSCLC after resistance to osimertinib who received osimertinib combined with anlotinib as third‐line and post‐third‐line treatments between June 2019 and March 2022 were retrospectively collected through the electronic medical record system of the Cancer Hospital, Chinese Academy of Medical Sciences and Peking Union Medical College.

### Inclusion criteria

(1) Age ≥18 years with an Eastern Cooperative Oncology Group Performance Status (ECOG PS) score of 0–2.

(2) Cytological or histological diagnosis of NSCLC.

(3) Stage III/IV according to the American Joint Committee on Cancer (AJCC) stage of lung cancer.

(4) With EGFR sensitive mutation (exon 19 del and exon 21 L858R) and EGFR T790M mutations, acquiring drug resistance after receiving a first‐ or second‐generation EGFR‐TKI, then acquiring drug resistance after receiving osimertinib.

(5) Having at least one measurable lesion, evaluated for clinical efficacy according to the Response Evaluation Criteria in Solid Tumors (RECIST) version 1.1.

(6) No contraindications to anlotinib found in clinical routine examination prior to treatment.

(7) Life expectancy of 3 months or more.

### Exclusion criteria

(1) Severe functional impairment of heart, lung, kidney, or other important organs.

(2) Obvious symptoms of hemoptysis or hemoptysis volume >50 ml/d.

(3) Coagulation disorders and other bleeding diseases.

(4) Pregnant or lactating women.

(5) Incomplete clinical data and lack of follow‐up information.

### Therapeutic regimen

Patients meeting the inclusion criteria were given 80 mg of osimertinib orally once a day, combined with anlotinib orally. The initial dosage of anlotinib was 12 mg, and anlotinib was stopped for 1 week after 2 weeks of continuous medication. Patients received anlotinib every 3 weeks as a treatment cycle until disease progressed or adverse events could not be tolerated. Dose was adjusted or stopped depending on the adverse events.

### Assessment of clinical efficacy

According to RECIST 1.1, all patients were evaluated for clinical efficacy and safety by magnetic resonance imaging (MRI) and/or computed tomography (CT), hematologic tumor indicators, and other conditions every two cycles(every 6 weeks). Primary endpoints were progression‐free survival (PFS) and overall survival (OS). Secondary endpoints were the objective response rate (ORR) and the disease control rate (DCR). The follow‐up period was up to March 2022.

### Evaluation criteria for adverse events

The grade of adverse events in patients treated with osimertinib combined with anlotinib was evaluated and the incidence of severe adverse events (grade 3 and above) was counted according to Common Terminology Criteria for Adverse Events (CTCAE) 5.0.

### Statistical method

SPSS 25.0 statistical software was used for statistical analysis. Kaplan–Meier and log‐rank tests were used for survival analysis and survival curve. Univariate and multivariate analyses was conducted by the Cox regression model. The value *p* < 0.05 was defined as statistically significant.

## RESULTS

### Baseline characteristics

A total of 34 patients (14 males and 20 females) meeting the inclusion criteria was collected. The median age was 65 years, of which 21 patients were 65 years or older. There were four former smokers and 30 nonsmokers. The ECOG PS score was 0–1 in 30 cases, 2 in four cases, EGFR exon 19 deletion occurred in 16 patients, and EGFR exon 21 L858R in 18 patients. There were eight patients with a family history of cancer, 21 patients with primary diseases (such as hypertension, diabetes, coronary heart disease, cerebral infarction, etc.), 24 patients had a history of chemotherapy, eight patients had a history of receiving bevacizumab, eight patients had a history of thoracic radiotherapy, 12 patients had a history of surgery, 16 patients had brain metastases, eight patients ha bone metastasis, three patients had liver metastasis, eight patients had pleural metastasis, and six patients had malignant pleural effusion. The baseline characteristics are shown in Table 1.

**TABLE 1:** Clinical characteristics of 34 non‐small‐cell lung cancer patients

Characteristic	Total (*n*)	Proportion (%)	mPFS (m)	*χ* ^2^ value	*p*
Sex					
Male	14	41.2%	7.0	1.224	0.269
Female	20	58.8%	6.0		
Age					
≥65	21	61.8%	6.0	0.885	0.347
<65	13	38.2%	7.0		
Smoking history					
Yes	4	11.8%	7.0	0.509	0.476
No	30	88.2%	6.0		
ECOG PS					
0–1	30	88.2%	7.0	14.342	0.000*
2	4	11.8%	2.0		
EGFR genotype					
exon 19 del	16	47.1%	7.0	0.929	0.335
exon 21 L858R	18	52.9%	4.0		
Family history					
Yes	8	23.5%	6.0	0.000	0.995
No	26	76.5%	6.0		
Primary disease					
Yes	21	61.8%	6.0	0.062	0.804
No	13	38.2%	6.0		
Prior chemotherapy					
Yes	24	70.6%	6.0	0.542	0.462
No	10	29.4%	4.0		
Prior bevacizumab					
Yes	8	23.5%	6.0	0.011	0.917
No	26	76.5%	6.0		
Prior radiotherapy					
Yes	8	23.5%	12.0	4.698	0.030*
No	26	76.5%	5.0		
Prior operation					
Yes	12	35.3%	4.0	0.205	0.650
No	22	64.5%	6.0		
Brain metastases					
Yes	16	47.1%	4.0	4.380	0.036*
No	18	52.9%	7.0		
Bone metastases					
Yes	8	23.5%	4.0	5.195	0.023*
No	26	76.5%	7.0		
Liver metastases					
Yes	3	8.8%	2.0	2.626	0.105
No	31	91.2%	6.0		
Pleural metastasis					
Yes	8	23.5%	4.0	0.865	0.352
No	26	76.5%	6.0		
Malignant pleural effusion					
Yes	6	17.6%	4.0	3.613	0.057
No	28	82.4%	7.0		

*Abbreviations*: ECOG PS, eastern cooperative oncology group performance status; EGFR, epidermal growth factor receptor; mPFS, median progression‐free survival.

* *p* < 0.05.

### Clinical efficacy

The ORR and DCR of patients with advanced NSCLC treated with osimertinib combined with anlotinib as the third‐line and post‐third‐line treatments were 20.6% and 88.2%, including no cases with complete response (CR), seven cases with partial response (PR), 23 cases of stable disease (SD), and four cases of progressive disease (PD).

### Survival analysis

**FIGURE 1:**
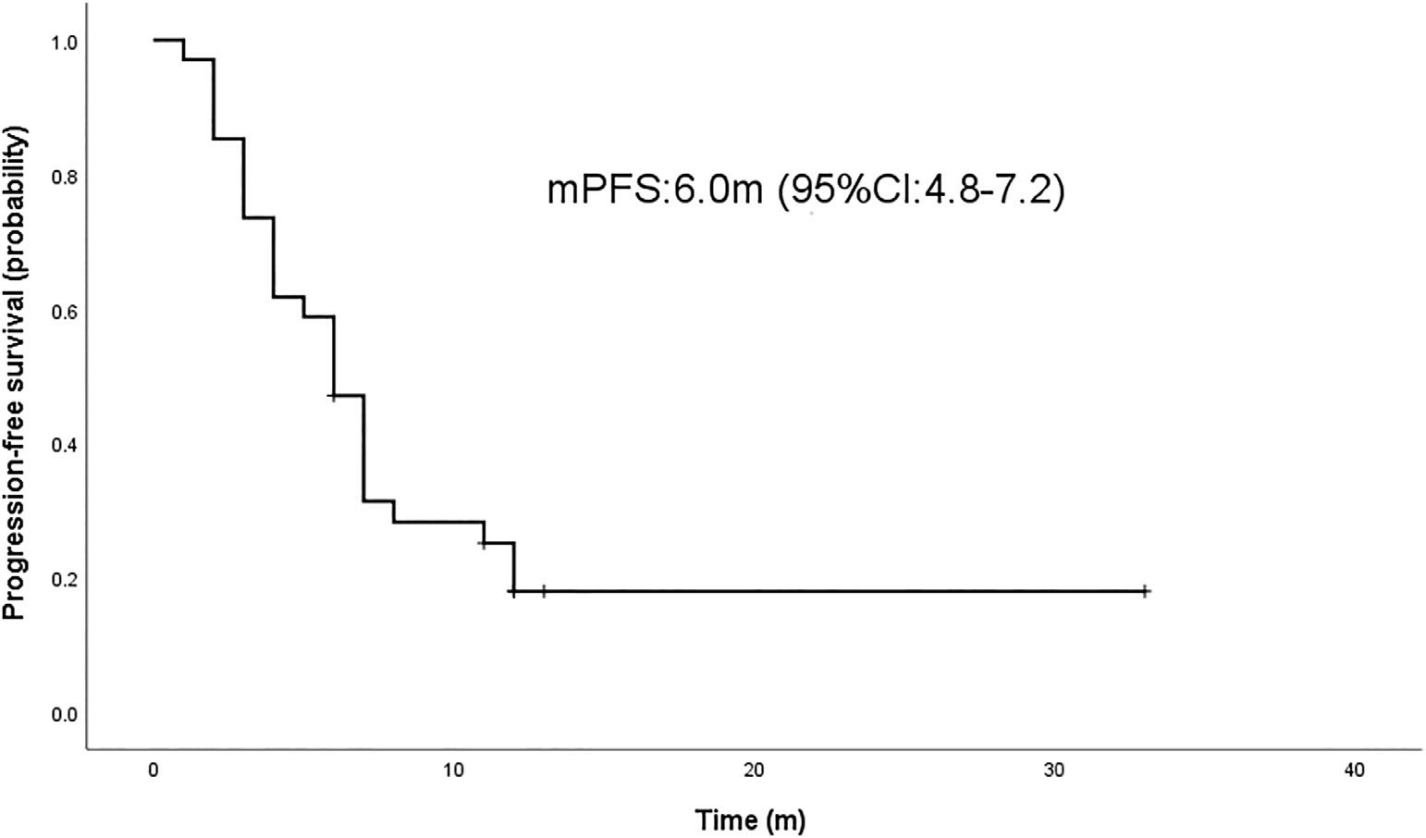
Median progression‐free survival (mPFS) curve of osimertinib plus anlotinib in the treatment

**FIGURE 2:**
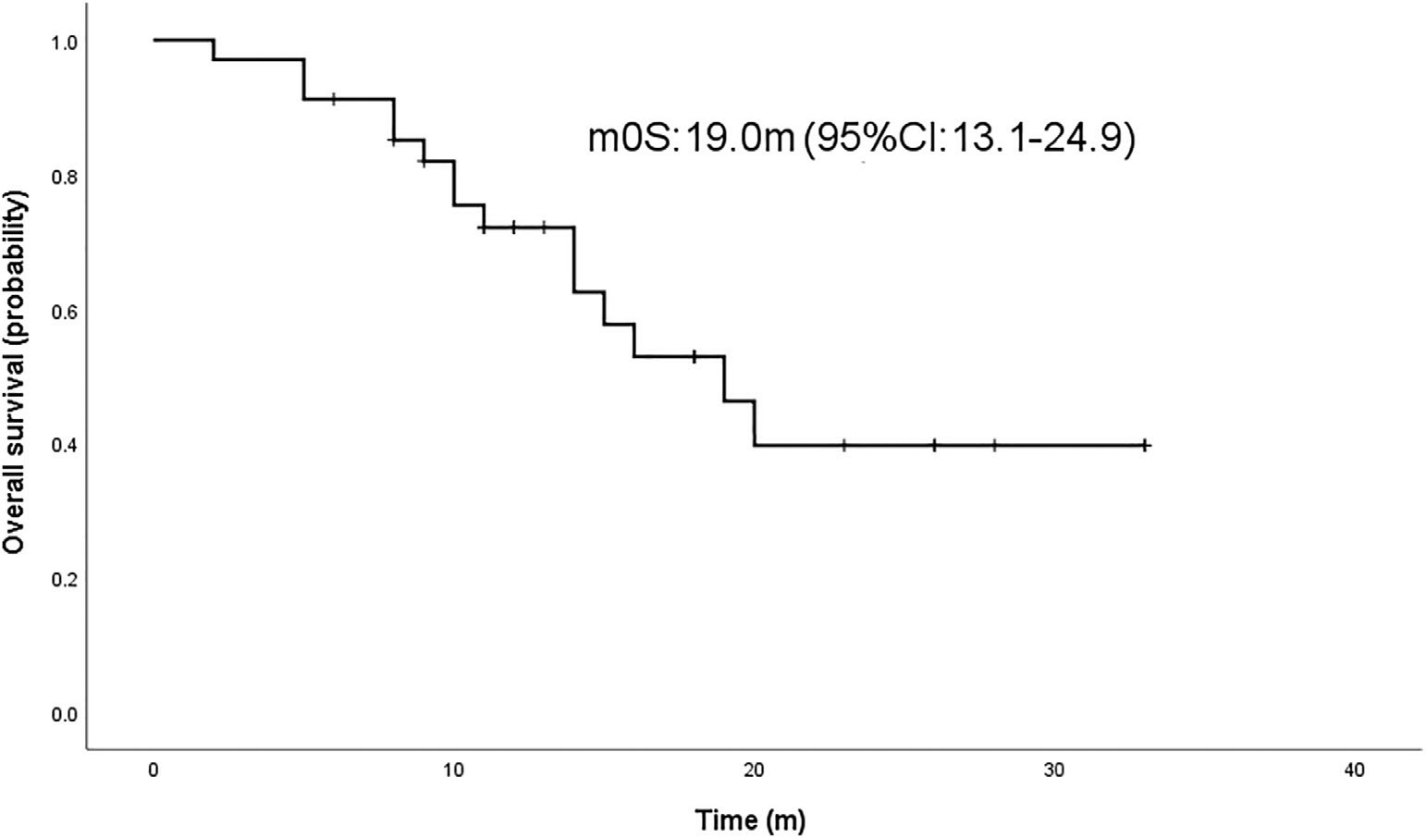
Median overall survival (mOS) curve of osimertinib plus anlotinib in the treatment

Survival analysis was performed by Kaplan–Meier to calculate the PFS and OS of osimertinib combined with anlotinib. The results showed the median progression‐free survival (mPFS) was 6.0 months (95% confidence interval [CI] 4.8–7.2) and median overall survival (mOS) was 19.0 months (95% CI 13.1–24.9) for osimertinib plus anlotinib (Figures 1 and 2).

### Safety evaluation

Toxicity was assessed in all patients. The most common adverse events were grade 1–2, including hand and foot syndrome, hemoptysis, epistaxis, hypertension, general fatigue, vomiting, diarrhea, weight loss, proteinuria, etc. A total of three cases (11.1%) of grade 3 adverse events occurred, including one case of grade 3 hypertension, which improved after drug withdrawal and antihypertensive treatment. Grade 3 vomiting occurred in one case, and the symptoms were improved after using antiemetics and gastric protection. Grade 3 proteinuria occurred in one case and gradually improved after drug withdrawal. No grade 4 or above adverse events occurred (Table 2).

**TABLE 2:** Adverse events for osimertinib plus anlotinib in the treatment

Adverse event	All grade		1–2 grade		3 grade		4 grade	
	*N*	%	*N*	%	*N*	%	*N*	%
Hand‐foot syndrome	1.0	3.7	1.0	3.7	0.0	0.0	0.0	0.0
Hemoptysis	2.0	7.4	2.0	7.4	0.0	0.0	0.0	0.0
Epistaxis	3.0	11.1	3.0	11.1	0.0	0.0	0.0	0.0
Hypertension	5.0	18.6	4.0	14.9	0.0	3.7	0.0	0.0
Weakness	2.0	7.4	2.0	7.4	0.0	0.0	0.0	0.0
Vomiting	1.0	3.7	0.0	0.0	1.0	3.7	0.0	0.0
Diarrhea	5.0	18.6	5.0	18.6	0.0	0.0	0.0	0.0
Proteinuria	5.0	18.6	4.0	14.9	1.0	3.7	0.0	0.0
Thrombocytopenia	1.0	3.7	1.0	3.7	0.0	0.0	0.0	0.0
Weight loss	1.0	3.7	1.0	3.7	0.0	0.0	0.0	0.0
Oral mucositis	1.0	3.7	1.0	3.7	0.0	0.0	0.0	0.0

### Subgroup analysis

The factors that might influence the efficacy of 34 patients receiving osimertinib combined with anlotinib were analyzed by log rank test. The results showed that there were no significant differences in mPFS among patients with different sex, age, EGFR genotype, family history, smoking history, history of receiving chemotherapy or bevacizumab, surgery, liver metastasis, pleural metastasis, and malignant pleural effusion. The mPFS of ECOG PS 0–1 group and ECOG PS 2 group were 7.0 months and 2.0 months, respectively, and there was a statistical difference between the two groups (*p* < 0.05). The mPFS of patients with and without thoracic radiotherapy history were 12.0 and 5.0 months, respectively, and there was a statistical difference between the two groups (*p* < 0.05). The mPFS of patients with and without brain metastases were 4.0 months and 7.0 months, respectively, and there was a statistical difference between the two groups (*p* < 0.05). The mPFS of patients with and without bone metastasis were 4.0 months and 7.0 months, respectively, and the results were statistically different between the two groups (*p* < 0.05) (Table 1).

### Prognostic factors

Univariate analysis showed that ECOG PS score and bone metastasis were significantly correlated with prognosis (*p* < 0.05). Multivariate Cox regression analysis was performed for the prognostic factors in univariate analysis. The results showed that ECOG PS score and bone metastasis were independent prognostic factors affecting the survival outcomes of advanced NSCLC after resistance to osimertinib combined with anlotinib (Table 3).

**TABLE 3:** Univariate and multivariate analysis of PFS of osimertinib plus anlotinib in the treatment

Variate	Univariate analysis		Multivariate analysis	
	HR (95% CI)	*p* value	HR (95% CI)	*p* value
Age	1.427 (0.640–3.182)	0.385		
Sex	1.512 (0.683–3.346)	0.307		
Smoking history	0.667 (0.199–2.238)	0.513		
ECOG PS	6.767 (2.060–22.237)	0.002*	7.207 (2.110–24.619)	0.002
EGFR genotype	1.144 (0.781–1.676)	0.489		
Family history	0.997 (0.401–2.480)	0.995		
Primary disease	0.912 (0.416–1.998)	0.818		
Prior chemotherapy	1.375 (0.551–3.434)	0.495		
Prior bevacizumab	0.959 (0.383–2.384)	0.923		
Prior radiotherapy	0.350 (0.120–1.020)	0.054		
Prior operation	0.842 (0.377–1.880)	0.674		
Brain metastases	2.182 (0.981–4.851)	0.056		
Bone metastases	2.467 (1.040–5.851)	0.040*	2.541 (1.061–6.083)	0.036
Liver metastases	2.467 (0.726–8.383)	0.148		
Pleural metastases	0.653 (0.247–1.729)	0.391		
Malignant pleural effusion	2.303 (0.895–5.928)	0.084		

*Abbreviations*: CI, confidence interval; ECOG PS, eastern cooperative oncology group performance status; EGFR, epidermal growth factor receptor; HR, hazard ratio.

* *p* < 0.05.

## DISCUSSION

Osimertinib was initially approved for treatment of EGFR driver‐positive patients exhibiting a T790M resistance mutation in the second‐line setting and is now emerging as the new standard of care for all EGFR mutation patients as the first‐line treatment. However, the issue of acquired drug resistance has emerged. The resistance mechanisms are being explored, mainly EGFR‐dependent and EGFR‐independent types, of which mesenchymal‐epithelial transition factor (MET) amplification and C797S mutation are the two most common resistance mechanisms.^8^ Currently, platinum‐based combination chemotherapy is the recommended standard of care,^9^ but ongoing clinical trials have provided some new possibilities,^10^ for example targeted drug MET inhibitors,^11^ EGFR‐TKIs in combination with chemotherapy,^12^ chemotherapy in combination with immune checkpoint inhibitor (ICI)^13^ and VEGFR inhibitors.^14^

Angiogenesis plays a crucial role in tumor growth and metastasis, therefore blocking the angiogenesis pathway has become a clinical strategy for cancer treatment.^15^ Meanwhile, the EGFR and VEGFR signaling pathways are closely related and have crossover and synergistic effects.^16^ For example, activation of EGFR can promote secretion of VEGF, while inhibition of the EGFR and VEGFR signaling pathways may have a synergistic effect in NSCLC with EGFR sensitive mutations.^17^ Therefore, EGFR‐TKI combined with VEGFR inhibitors also provides a new option for treatment after resistance to osimertinib. A study by Helena et al. showed that in advanced NSCLC patients with EGFR mutation who had not received EGFR‐TKIs and/or VEGFR inhibitors, the 12‐month rate of PFS was 76% and mPFS was 19 months when receiving osimertinib combined with bevacizumab.^18^ A multicenter randomized controlled phase II study of 81 patients with EGFR T90M mutation in NSCLC, including 41 patients receiving osimertinib and 40 patients receiving osimertinib plus bevacizumab, showed that the ORR of osimertinib plus bevacizumab was superior to that of osimertinib monotherapy(68% vs. 54%). Although osimertinib plus bevacizumab did not prolong PFS and OS compared with osimertinib alone,^19^ there was no increase in toxicity in the combination therapy compared with monotherapy. In a multicenter retrospective study in China, a total of 39 patients with lung adenocarcinoma who were resistant to osimertinib combined with apatinib were included, and the results showed that ORR, DCR, and mPFS were 12.8%, 79.5%, and 4 months, respectively.^20^

Anlotinib is a novel oral multitarget TKI targeting VEGFR‐2 and VEGFR−3, FGFR1‐4, PDGFR‐α, PDGFR‐β, and c‐Kit, thereby inhibiting tumor growth and angiogenesis.^21^ The ALTER 0303 trial showed that patients with advanced NSCLC receiving three or more lines of treatment with anlotinib had significantly better OS (9.6 months vs. 6.3 months) and PFS (5.4 months vs. 1.4 months) than patients receiving placebo. In May 2018, anlotinib was approved by the China Food and Drug Administration as a third‐line treatment for refractory advanced NSCLC.^22^ Li et al. found that anlotinib combined with gefitinib could inhibit the proliferation of NSCLC cells in vitro and tumor angiogenesis in vivo. In this study, 20 patients with NSCLC who were resistant to gefitinib or erlotinib were enrolled and then received gefitinib or erlotinib combined with anlotinib, and the results showed that the mPFS was 15.7 months. Meanwhile, in the nude mouse model, EGFR‐TKI combined with anlotinib significantly inhibited the proliferation of lung cancer cells.^23^ A similar conclusion was drawed in a retrospective study by Zhang et al., who found that in a gefitinib‐resistant lung adenocarcinoma model, anlotinib reversed resistance to gefitinib by enhancing the antiproliferation and pro‐apoptotic effects of gefitinib. Gefitinib combined with anlotinib exerted a synergistic antitumor effect by downregulating the activation of VEGFR2 and its downstream effectors (Akt and ERK). Among the 24 NSCLC patients with acquired EGFR‐TKI resistance, the ORR and DCR of EGFR‐TKI combined with anlotinib were 20.8% and 95.8%, respectively. The mPFS was 11.53 ± 2.41 months and mOS was not reached.^24^ However, at present, there are few studies on the combination of osimertinib and anlotinib after resistance to osimertinib. Zhou et al. reported a case of a patient with advanced lung adenocarcinoma harboring resistance to Osimertinib who reached PR for 9 months when treated with osimertinib combined with chemotherapy and anlotinib, and was then treated with osimertinib and anlotinib as maintain therapy.^25^ In a retrospective study conducted by Zhou et al., 33 patients with advanced NSCLC who were resistant to osimertinib as the first‐line treatment were enrolled. The results showed that after osimertinib combined with anlotinib, one patient achieved CR, meanwhlie ORR and DCR were 81.8% and 97.0%, respectively. The mPFS was 15.5 months and mOS was 23.8 months.^26^

In this study, the ORR and DCR of osimertinib‐resistant NSCLC patients treated with osimertinib combined with anlotinib were 20.6% and 88.2%, respectively. In terms of safety, the retrospective analysis of the patients in this study showed that there were three cases of grade 3 adverse events (hypertension, vomiting, and proteinuria), which were gradually controlled after drug withdrawal or symptomatic treatment. No grade 4 or above adverse events were observed. These adverse events may be related to drug characteristics and individual heterogeneity,^27^ therefore the toxicity of osimertinib combined with anlotinib for third‐line treatment and post‐third‐line treatment are controllable.

The ECOG PS score is one of the most important independent prognostic factors for a variety of tumors, including advanced NSCLC, as an important way of measuring patients' physical condition and tolerance to treatment based on their self‐care ability, daily activity ability, and walking and working energy.^28^ A meta‐analysis showed that for NSCLC patients receiving second‐line chemotherapy, patients with an ECOG PS score of 2 had worse OS and HR of 3.01 (95% CI 2.41–3.76) compared with patients with an ECOG PS score of 0. Another meta‐analysis showed that patients with ECOG PS ≥2 had poor OS, PFS, and ORR, and therefore concluded that ECOG PS score is a prognostic factor for advanced NSCLC receiving immunotherapy.^29^ In a retrospective study by Li et al., among patients with advanced NSCLC who received post‐third‐line treatment with anlotinib, patients with ECOG PS score ≥2 had poorer OS and HR of 1.37 (95% CI 0.65–2.87) compared with those with ECOG PS 0–1.^30^ In this study, the mPFS of patients with ECOG PS 0–1 and 2 scores were 7.0 and 2.0 months, respectively (*p* < 0.05). Meanwhile, multivariate Cox regression showed that ECOG PS was an independent prognostic factor (*p* < 0.05), therefore patients with a good ECOG PS score may be more likely to benefit from osimertinib combined with anlotinib, possibly because patients with a bad ECOG PS score have poor tolerance to treatment, thus affecting clinical efficacy.

Bone is one of the common metastatic sites of lung cancer, with about 20–40% of NSCLC patients having bone metastasis,^31^ and lung cancer patients with bone metastasis tend to have a poor prognosis. Studies have shown that the 3‐year OS of NSCLC patients with bone metastasis decreased from 71.6% to 46.8%.^32^ In a retrospective study to explore the prognostic value of serological inflammatory biomarkers in the treatment of anlotinib, multivariate Cox regression analysis showed that patients with bone metastases were associated with poor PFS. Although this negative association did not reach a significant difference,^33^ it suggested that patients with NSCLC with bone metastases had poor clinical outcomes. In our study, the mOS of eight patients with bone metastasis was significantly lower than that of 26 patients without bone metastasis (8.0 months vs. 18.0 months, *p* < 0.05). Multivariate Cox regression analysis showed that bone metastasis was an independent factor of prognosis (*p* < 0.05). It was observed that the ECOG PS score of patients with bone metastasis was ≥1, suggesting that the mPFS of patients with bone metastasis was significantly lower than in those without bone metastasis, which may be due to weak physical condition and poor tolerance to treatment. Meanwhile, we found that seven of eight patients with bone metastasis were accompanied by metastasis to other sites or even multiple metastases. This could also explain why NSCLC patients with bone metastases have poorer survival benefits.

In summary, this study observed and analyzed the efficacy and safety of osimertinib combined with anlotinib in three or more lines of treatment of 34 patients with advanced NSCLC who harbored T90M mutations after resistance to first‐ or second‐generation EGFR‐TKI. It was concluded that osimertinib combined with anlotinib was effective and the adverse events were tolerated. However, our study also has some limitations: the sample size was relatively small and there was no control group, which may lead to bias. Future multicenter, larger or prospective, randomized controlled studies should be performed for further exploration.

## AUTHOR CONTRIBUTIONS

Corresponding authors had full access to the data in the study and all authors take responsibility for the integrity of the data and the accuracy of the data analysis. Study concept and design: M.W. and Y.L. Acquisition of data: M.W., X.H., L.W., and T.C. Analysis and interpretation of the data: M.W., J.Z., and T.C. Drafting of the manuscript: M.W., J.Z., and Y.L. Critical revision of the manuscript for important intellectual content: Y. S. and Y.L.

## CONFLICT OF INTEREST STATEMENT

The authors declare that there are no conflicts of interest.

## References

[1] Herbst RS , Morgensztern D , Boshoff C . The biology and management of non‐small cell lung cancer. Nature. 2018;553(7689):446–54. 29364287 10.1038/nature25183

[2] Inoue A . Progress in individualized treatment for EGFR‐mutated advanced non‐small cell lung cancer. Proc Jpn Acad Ser B Phys Biol Sci. 2020;96(7):266–72. 10.2183/pjab.96.020 PMC7443375 32788550

[3] Papadimitrakopoulou VA , Mok TS , Han JY , Ahn MJ , Delmonte A , Ramalingam SS , et al. Osimertinib versus platinum–pemetrexed for patients with EGFR T790M advanced NSCLC and progression on a prior EGFR‐tyrosine kinase inhibitor: AURA3 overall survival analysis. Ann Oncol. 2020;31(11):1536–44. 32861806 10.1016/j.annonc.2020.08.2100

[4] Soria JC , Ohe Y , Vansteenkiste J , Reungwetwattana T , Chewaskulyong B , Lee KH , et al. Osimertinib in untreated EGFR‐mutated advanced non–small‐cell lung cancer. N Engl J Med. 2018;378(2):113–25. 29151359 10.1056/NEJMoa1713137

[5] Mok TS , Wu YL , Ahn MJ , Garassino MC , Kim HR , Ramalingam SS , et al. Osimertinib or platinum–pemetrexed in EGFR T790M–positive lung cancer. N Engl J Med. 2017;376(7):629–40. 27959700 10.1056/NEJMoa1612674 PMC6762027

[6] Larsen AK , Ouaret D , El Ouadrani K , Petitprez A . Targeting EGFR and VEGF (R) pathway cross‐talk in tumor survival and angiogenesis. Pharmacol Ther. 2011;131(1):80–90. 21439312 10.1016/j.pharmthera.2011.03.012

[7] Cheng Y , Wang Q , Li K , Shi J , Liu Y , Wu L , et al. Anlotinib vs placebo as third‐or further‐line treatment for patients with small cell lung cancer: a randomised, double‐blind, placebo‐controlled phase 2 study. Br J Cancer. 2021;125(3):366–71. 34006926 10.1038/s41416-021-01356-3 PMC8329046

[8] Schmid S , Li JJN , Leighl NB . Mechanisms of osimertinib resistance and emerging treatment options. Lung Cancer. 2020;147:123–9. 32693293 10.1016/j.lungcan.2020.07.014

[9] Oxnard GR , Hu Y , Mileham KF , Husain H , Costa DB , Tracy P , et al. Assessment of resistance mechanisms and clinical implications in patients with EGFR T790M–positive lung cancer and acquired resistance to osimertinib. JAMA Oncol. 2018;4(11):1527–34. 30073261 10.1001/jamaoncol.2018.2969 PMC6240476

[10] Wu L , Ke L , Zhang Z , Yu J , Meng X . Development of EGFR TKIs and options to manage resistance of third‐generation EGFR TKI osimertinib: conventional ways and immune checkpoint inhibitors. Front Oncol. 2020;10:602762. 33392095 10.3389/fonc.2020.602762 PMC7775519

[11] Sequist LV , Han JY , Ahn MJ , Cho BC , Yu H , Kim SW , et al. Osimertinib plus savolitinib in patients with EGFR mutation‐positive, MET‐amplified, non‐small‐cell lung cancer after progression on EGFR tyrosine kinase inhibitors: interim results from a multicentre, open‐label, phase 1b study. Lancet Oncol. 2020;21(3):373–86. 32027846 10.1016/S1470-2045(19)30785-5

[12] Okada M , Tanaka K , Asahina H , Harada T , Hamai K , Watanabe K , et al. Safety analysis of an open label, randomized phase 2 study of osimertinib alone versus osimertinib plus carboplatin‐pemetrexed for patients with non–small cell lung cancer (NSCLC) that progressed during prior epidermal growth factor receptor (EGFR) tyrosine kinase inhibitor (TKI) therapy and which harbors a T790M mutation of EGFR. J Clin Oncol. 2018;36(15_suppl):e21073–3.

[13] Yang B , Long Y , Hu Y . Chemoimmunotherapy combination: potential option for metastatic NSCLC patients with EGFR mutation resistant to osimertinib. J Clin Oncol. 2019;37(15_suppl):e20618–8.

[14] Socinski MA , Mok TS , Nishio M , Jotte RM , Cappuzzo F , Orlandi F , et al. Abstract CT216: IMpower150 final analysis: efficacy of atezolizumab (atezo) + bevacizumab (bev) and chemotherapy in first‐line (1L) metastatic nonsquamous (nsq) non‐small cell lung cancer (NSCLC) across key subgroups. Cancer Res. 2020;80(16_Supplement):CT216‐CT216.

[15] Gao Y , Liu P , Shi R . Anlotinib as a molecular targeted therapy for tumors. Oncol Lett. 2020;20(2):1001–14. 32724339 10.3892/ol.2020.11685 PMC7377159

[16] Karaman S , Leppänen VM , Alitalo K . Vascular endothelial growth factor signaling in development and disease. Development. 2018;145(14):151019. 10.1242/dev.151019 30030240

[17] Medina G , Vera‐Lastra O , Peralta‐Amaro AL , Jiménez‐Arellano MP , Saavedra MA , Cruz‐Domínguez MP , et al. Metabolic syndrome, autoimmunity and rheumatic diseases. Pharmacol Res. 2018;133:277–88. 29382608 10.1016/j.phrs.2018.01.009

[18] Helena AY , Schoenfeld AJ , Makhnin A , Kim R , Rizvi H , Tsui D , et al. Effect of osimertinib and bevacizumab on progression‐free survival for patients with metastatic EGFR‐mutant lung cancers: a phase 1/2 single‐group open‐label trial. JAMA Oncol. 2020;6(7):1048–54. 32463456 10.1001/jamaoncol.2020.1260 PMC7256866

[19] Akamatsu H , Toi Y , Hayashi H , Fujimoto D , Tachihara M , Furuya N , et al. Efficacy of osimertinib plus bevacizumab vs osimertinib in patients with EGFR T790M–mutated non‐small cell lung cancer previously treated with epidermal growth factor receptor–tyrosine kinase inhibitor: West Japan oncology group 8715L phase 2 randomized clinical trial. JAMA Oncol. 2021;7(3):386–94. 33410885 10.1001/jamaoncol.2020.6758 PMC7791398

[20] Yang X , Xia Y , Xu L , et al. Efficacy and safety of combination treatment with apatinib and osimertinib after osimertinib resistance in epidermal growth factor receptor‐mutant non‐small cell lung carcinoma – a retrospective analysis of a multicenter clinical study. Front Mol Biosci. 2021;8:639892–2. 34026823 10.3389/fmolb.2021.639892 PMC8131525

[21] Syed YY . Anlotinib: first global approval. Drugs. 2018;78(10):1057–62. 29943374 10.1007/s40265-018-0939-x

[22] Han B , Li K , Wang Q , Zhang L , Shi J , Wang Z , et al. Effect of anlotinib as a third‐line or further treatment on overall survival of patients with advanced non‐small cell lung cancer: the ALTER 0303 phase 3 randomized clinical trial. JAMA Oncol. 2018;4(11):1569–75. 30098152 10.1001/jamaoncol.2018.3039 PMC6248083

[23] Li T , Qian Y , Zhang C , Uchino J , Provencio M , Wang Y , et al. Anlotinib combined with gefitinib can significantly improve the proliferation of epidermal growth factor receptor‐mutant advanced non‐small cell lung cancer in vitro and in vivo. Transl Lung Cancer Res. 2021;10(4):1873–88. 34012799 10.21037/tlcr-21-192 PMC8107735

[24] Zhang C , Cao H , Cui Y , Jin S , Gao W , Huang C , et al. Concurrent use of anlotinib overcomes acquired resistance to EGFR‐TKI in patients with advanced EGFR‐mutant non‐small cell lung cancer. Thorac Cancer. 2021;12(19):2574–84. 34510760 10.1111/1759-7714.14141 PMC8487816

[25] Zhou R , Song L , Zhang W , Shao L , Li X , Li X . Combination of osimertinib and anlotinib may overcome the resistance mediated by in cis EGFR T790M‐C797S in NSCLC: a case report. Onco Targets Ther. 2021;28(14):2847–51. 10.2147/OTT.S298655 PMC8093742 33958875

[26] Zhou B , Gong Q , Li B , Qie HL , Li W , Jiang HT , et al. Clinical outcomes and safety of osimertinib plus anlotinib for patients with previously treated EGFR T790M‐positive NSCLC: a retrospective study. J Clin Pharm Ther. 2022;47(5):643–51. 35023208 10.1111/jcpt.13591

[27] Sun Y , Niu W , Du F , Du C , Li S , Wang J , et al. Safety, pharmacokinetics, and antitumor properties of anlotinib, an oral multi‐target tyrosine kinase inhibitor, in patients with advanced refractory solid tumors. J Hematol Oncol. 2016;9(1):1–9. 27716285 10.1186/s13045-016-0332-8 PMC5051080

[28] Albain KS , Crowley JJ , LeBlanc M , Livingston RB . Survival determinants in extensive‐stage non‐small‐cell lung cancer: the southwest oncology group experience. J Clin Oncol. 1991;9(9):1618–26. 1651993 10.1200/JCO.1991.9.9.1618

[29] Dall'Olio FG , Maggio I , Massucci M , Mollica V , Fragomeno B , Ardizzoni A . ECOG performance status ≥2 as a prognostic factor in patients with advanced non small cell lung cancer treated with immune checkpoint inhibitors—a systematic review and meta‐analysis of real world data. Lung Cancer. 2020;145:95–104. 32417680 10.1016/j.lungcan.2020.04.027

[30] Li L , Liu W , Wang Y , Zhang Q , Chi C , Bai Q , et al. Anlotinib as a post‐third‐line therapy for the treatment of advanced nonsmall cell lung cancer. J Chemother. 2021;33(7):492–8. 33818318 10.1080/1120009X.2021.1906036

[31] Kuchuk M , Addison CL , Clemons M , Kuchuk I , Wheatley‐Price P . Incidence and consequences of bone metastases in lung cancer patients. J Bone Oncol. 2013;2(1):22–9. 26909268 10.1016/j.jbo.2012.12.004 PMC4723355

[32] Yang HL , Liu T , Wang XM , Xu Y , Deng SM . Diagnosis of bone metastases: a meta‐analysis comparing 18FDG PET, CT, MRI and bone scintigraphy. Eur Radiol. 2011;21(12):2604–17. 21887484 10.1007/s00330-011-2221-4

[33] Chen R , Lu FY , Liu B , Huang J , Zhou M , Dai R , et al. Absolute neutrophil count in the peripheral blood predicts prognosis in lung cancer patients treated with anlotinib. Cancer Manag Res. 2021;13:3619–27. 33976572 10.2147/CMAR.S307368 PMC8106457

